# Exploring *hTERT* promoter methylation in cutaneous T‐cell lymphomas

**DOI:** 10.1002/1878-0261.12946

**Published:** 2021-10-12

**Authors:** Alain Chebly, Joana Ropio, Jean‐Marie Peloponese, Sandrine Poglio, Martina Prochazkova‐Carlotti, Floriane Cherrier, Jacky Ferrer, Yamina Idrissi, Evelyne Segal‐Bendirdjian, Eliane Chouery, Chantal Farra, Anne Pham‐Ledard, Marie Beylot‐Barry, Jean‐Philippe Merlio, Roland Tomb, Edith Chevret

**Affiliations:** ^1^ INSERM BaRITOn U1053 University of Bordeaux France; ^2^ Medical Genetics Unit (UGM) Faculty of Medicine Saint Joseph University Beirut Lebanon; ^3^ Cancer Biology Group Institute of Biomedical Sciences of Abel Salazar Instituto de Investigação e Inovação em Saúde Institute of Molecular Pathology and Immunology (Ipatimup) Porto University Portugal; ^4^ CNRS IRIM‐UMR 9004 Research Institute in Infectiology of Montpellier University of Montpellier France; ^5^ INSERM UMR‐S 1124, Team: Cellular Homeostasis Cancer and Therapies Université de Paris France; ^6^ Genetics Department Faculty of Medicine Hotel Dieu de France Medical Center Beirut Lebanon; ^7^ Dermatology Department Bordeaux University Hospital Center France; ^8^ Tumor Bank and Tumor Biology Laboratory Bordeaux University Hospital Center Pessac France; ^9^ Dermatology Department Faculty of Medicine Saint Joseph University Beirut Lebanon; ^10^ Present address: Department of Human Genetics Gilbert and Rose‐Marie Chagoury School of Medicine Lebanese American University Byblos Lebanon

**Keywords:** cutaneous T‐cell lymphomas, DNA methylation, DNMTi, HDACi, telomerase, TERT

## Abstract

Cutaneous T‐cell lymphomas (CTCLs) are telomerase‐positive tumors expressing *hTERT*, although neither gene rearrangement/amplification nor promoter hotspot mutations could explain the *hTERT* re‐expression. As the *hTERT* promoter is rich in CpG, we investigated the contribution of epigenetic mechanisms in its re‐expression. We analyzed *hTERT* promoter methylation status in CTCL cells compared with healthy cells. Gene‐specific methylation analyses revealed a common methylation pattern exclusively in tumor cells. This methylation pattern encompassed a hypermethylated distal region from −650 to −150 bp and a hypomethylated proximal region from −150 to +150 bp. Interestingly, the hypermethylated region matches with the recently named *TERT* hypermethylated oncogenic region (THOR). THOR has been associated with telomerase reactivation in many cancers, but it has so far not been reported in cutaneous lymphomas. Additionally, we assessed the effect of THOR on two histone deacetylase inhibitors (HDACi), romidepsin and vorinostat, both approved for CTCL treatment and a DNA methyltransferase inhibitor (DNMTi) 5‐azacytidine, unapproved for CTCL. Contrary to our expectations, the findings reported herein revealed that THOR methylation is relatively stable under these epigenetic drugs' pressure, whereas these drugs reduced the *hTERT* gene expression.

## Graphical abstract

Cutaneous T‐cell lymphomas (CTCLs) represent a group of lymphoproliferative disorders arising from the skin. CTCLs are characterized by *hTERT* gene expression despite the lack of *hTERT* amplifications or rearrangements. Here, we investigated *hTERT* promoter methylation and associated TERT hypermethylated oncogenic region (THOR) with *hTERT* reactivation in CTCL. Additionally, we evaluated THOR methylation and *hTERT* expression after treatment with epigenetic drugs. Altogether, our study offers a better understanding of the response to epigenetic drugs in patients with CTCL.

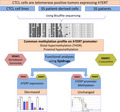

**Abbreviations**
CTCLs: cutaneous T‐cell lymphomas
DNMTi: DNA methyltransferase inhibitors
E‐box: enhancer box
HDACi: histone deacetylase inhibitors
hTERT: human telomerase reverse transcriptase gene
MZF‐2: myeloid zinc finger protein 2
NTC: nontreated cells
SS PDCs: Sézary syndrome patient‐derived cells
SS: Sézary syndrome
TBP: TATA‐box‐binding protein
TF: transcription factor
THOR: TERT hypermethylated oncogenic region
T‐MF: transformed tumor stage mycosis fungoïdes
TSS: transcription start site
WT1: Wilms' tumor 1

## 1 Introduction

Cutaneous T‐cell lymphomas (CTCLs) encompass a heterogeneous group of rare T lymphoproliferative disorders, characterized by clonal proliferation of malignant T cells involving the skin as a primary site. They include cutaneous anaplastic large cell lymphoma (C‐ALCL), mycosis fungoïdes (MF), and Sézary syndrome (SS) [1]. While most C‐ALCL and MF have an indolent course, some MF may progress to a transformed tumor stage (T‐MF) of poor prognosis. SS can be developed in a patient affected many years with MF, but it arises more frequently as erythroderma associated with a frank leukemic variant [2]. Treatment of MF/SS can be very challenging, especially in the advanced stages of the disease. The choice of the therapeutic agent is stage‐dependent, including drugs such as bexarotene, methotrexate, interferon‐alpha, and histone deacetylases inhibitors (HDACi) or the recently introduced monoclonal antibodies such as mogamulizumab, brentuximab vedotin, or IPH4102. While chemotherapies only allow short‐lived responses, allogenic stem cell transplantation remains the only curative option [3, 4].

In cancer cells, replicative immortality can be acquired through telomerase reactivation driven by the human telomerase reverse transcriptase (*hTERT*) gene expression [5]. The *hTERT* gene can be upregulated either by genetic mechanisms (like promoter mutations and less frequently gene amplifications or rearrangements [6]) or by epigenetic mechanisms (DNA methylation, histone modifications, and noncoding RNA effects [7, 8, 9, 10, 11, 12]). The *hTERT* promoter methylation stimulated the interest of the scientific committee because it is ‘unusual’, comprising two oppositely methylated regions: A hypermethylated region located upstream the transcription start site (TSS) and a hypomethylated region flanking the TSS and exon 1 [7, 11, 13]. The upstream hypermethylated region was reported to be associated with *hTERT* expression in different types of tumors [7, 11, 14, 15] and was recently investigated in a large cohort of cancer patients and cell lines [16, 17]. Since this region is frequently observed hypermethylated and associated with telomerase reactivation in tumor cells, it was recently named TERT hypermethylated oncogenic region (THOR) [16]. Although this region was investigated in many cancers, it was not investigated in cutaneous lymphomas [16]. Within THOR, three transcription factors' binding sites are typically located: two transcriptional silencers WT1 (Wilms' tumor 1) and MZF‐2 (myeloid zinc finger 2), and one transcriptional enhancer c‐MYC that binds to an enhancer box (E‐box) [18].

In a previous work, our team reported that *hTERT* is expressed in CTCL despite the lack of *hTERT* amplifications or rearrangements [19]. In a complementary study, we stated the absence of *hTERT* hot spot promoter mutations in these types of tumors (A. Ropio, M. Prochazkova‐Carlotti, R. Batista, A. Pestana, J. Ferrer, A. Chebly, Y. Idrissi, D. Cappellen, C. Durães, P. Boaventura, J. Vinagre, L. Azzi‐Martin, J. Cabeçadas, M. Campos, M. Beylot‐Barry, M. Sobrinho‐Simões, J. P. Merlio, P. Soares, & E. Chevret, In preparation). Since little is known about the mechanisms underlying the methylation changes during tumorigenesis [20] and since no *hTERT* promoter epigenetic investigation was reported in CTCL, we present herein a pioneer exploration in this rare pathology. We evaluate THOR methylation status in CTCL cell lines and in SS patient‐derived cells in comparison with healthy cells (CD34^+^ and CD4^+^ lymphocytes). We explore THOR methylation under the pressure of a demethylating agent, unapproved for CTCL; we describe the effect of clinically approved HDAC inhibitors on THOR methylation status.

## 2 Materials and methods

### 2.1 Cell lines, SS patient‐derived cells, and cell culture

Five CTCL cell lines were studied: Myla (T‐MF) (kindly provided by K. Kaltoft, Denmark), HuT78 (SS) (ATCC, Molsheim, France), and Mac1, Mac2A, and Mac2B (C‐ALCL) (DSMZ, Braunschweig, Germany). Cells were cultured in RPMI 1640 medium (Gibco, Gaithersburg, MD, USA) supplemented with 10% of fetal bovine serum (Eurobio, Les Ulis, France) and 100 U·mL^−1^ of penicillin and streptomycin (Gibco).

Four SS patient‐derived cells (PDCs) obtained from four SS patients (patients 1 to 4) were also investigated. They were cultured as recently described by Poglio *et al*. [21].

Cell lines and PDC cultures were incubated at 37 °C in a humidified incubator with 5% CO_2_.

### 2.2 SS patients and tumor cell isolation

Ten SS patients, eight females and two males, with a median age of 69.5 years (range: 52–93), were recruited for this study. The diagnosis was established in accordance with the criteria of the WHO‐EORTC (World Health Organization and the European Organization for Research and Treatment of Cancer) [1]. All of them presented a B2 stage; eight, a T4 stage; one, a T3 stage; and one, a T2b stage. Samples from patients 1 to 4 were used to establish PDC as mentioned above. Samples from patients 5 to 10 were used to obtain fresh SS cells. Peripheral blood mononuclear cells (PBMCs) were isolated using Pancoll (Pan Biotech, Aidenbach, Germany). Clonal TCRvβ was determined using IOTest® Beta Mark TCRVβ Repertoire Kit (Beckman Coulter, Villepinte, France). Tumor cells were sorted either according to the TCRvβ using a BD FACSAria™ II Cell Sorter (BD Biosciences, Le Pont de Claix, France) or according to the CD4^+^, as mentioned in Ref. [21] (Fig. S1 shows the evaluation of tumor cells' proportion before and after cell sorting). This study was approved by the local ethics committee and was carried out in accordance with the standards set by the Declaration of Helsinki. All SS patients included in this study signed an informed consent.

### 2.3 Controls and healthy donors

Seven healthy age‐matched donors were recruited from the *Etablissement Français du Sang* (EFS) in Bordeaux (DC 2015 2412‐18PLER012). PBMCs were isolated from peripheral blood samples, using Pancoll (Pan Biotech). CD4^+^ cells were manually sorted using CD4 MicroBeads Human Kit (Miltenyi Biotec, Bergisch Gladbach, Germany) and separated into two pools: A (three donors) and B (four donors). Progenitor/stem cells (CD34^+^) were collected from 20 healthy donors at the EFS and pooled together.

### 2.4 Chemicals

Drugs included in this study were two HDACi used to treat CTCL: romidepsin and vorinostat (Euromedex, Souffelweyersheim, France) and a DNA methyltransferase inhibitor (DNMTi): 5‐azacytidine not approved for CTCL treatment. Based on previous reports [22, 23], 1 × 10^6^ SS PDCs (1, 2, 3, and 4) were exposed to 10 nm of romidepsin or 3 µm of vorinostat during 48 h. The Hut78 cell line and SS PDCs 2 and 3 were exposed to 3, 1.7, and 2.3 nm of 5‐azacytidine during 72 h, respectively.

### 2.5 DNA/RNA isolation and cDNA synthesis

Genomic DNA was extracted using Quick‐DNA Microprep Kit (ZYMO Research, Freiburg im Breisgau, Germany). Total RNA was isolated using the Direct‐zol™ RNA Miniprep Kit (ZYMO Research). DNA and RNA concentrations were measured using the NanoDrop ND‐1000 Spectrophotometer (NanoDrop Technologies Inc., Wilmington, DE, USA). cDNA was synthetized from 100 ng of RNA using the SuperScript II Reverse Transcriptase Kit (Invitrogen, ThermoFisher Scientific, Courtaboeuf, France).

### 2.6 Locus‐specific bisulfite sequencing

For methylation analyses, we used the standard bisulfite sequencing method. While this approach requires standard molecular biology apparatus, it generates reliable and consistent results in gene‐specific methylation studies. Also, compared to other global genomic bisulfite techniques, standard bisulfite sequencing method offers, in the region of interest, the ability to detect the methylation status cell by cell of all consecutive CpGs [24].

Genomic DNA was bisulfite‐converted using the EZ‐DNA Methylation Kit (ZYMO Research). The region from −650 to +150 bp relative to the transcription start site (TSS) of *hTERT* was amplified by PCR using GO‐Taq G2 Hot Start (Promega, Fitchburg, WI, USA). Primers were bisulfite‐specific and completely devoid of CpG sites as previously described [7, 11]. Forward and reverse primer sequences and PCR conditions are listed in Table S1. Amplicon lengths were verified, and PCR products were purified using MACHEREY‐NAGEL Extraction Kit (MACHEREY‐NAGEL, Düren, Germany). Purified amplicons were cloned into the p‐GEM‐T Easy Vector System I (Promega), and then, competent *Escherichia coli* (Promega) were transformed using the ligation product. Bacterial suspensions were enriched in SOC medium (New England Biolabs, Ipswich, MA, USA). Colonies were grown overnight on LB (Luria‐Bertani) Agar containing 32 μg·mL^−1^ Xgal, 120 μg·mL^−1^ IPTG, and 100 μg·mL^−1^ ampicillin. After white colonies' selection and checking of the DNA insertion by PCR, colonies were incubated overnight for enrichment in LB medium with 100 μg·mL^−1^ ampicillin at 37 °C under agitation. Plasmid DNA was isolated using Nucleospin Plasmid Kit (MACHEREY‐NAGEL). Each sample was performed in duplicate. Ten to 30 clones were extracted and sequenced. DNA sequences were analyzed using chromaspro Software (Technelysium, South Brisbane, Queensland, Australia), and bisulfite images were obtained using quma (Riken, Japan, http://quma.cdb.riken.jp) [25].

### 2.7 *hTERT* and *WT1* expression analysis by quantitative real‐time PCR

cDNAs were amplified by quantitative real‐time PCR (qRT‐PCR) using Takyon™ No Rox SYBR® MasterMix dttP Blue (Eurogentec, Angers, France), and the following primer sets were used: *hTERT* gene, forward primer: 5′‐GCATTGGAATCAGACAGCAC‐3′ and reverse primer: 5′‐CCACGACGTAGTCCATGTTC‐3′, and housekeeping gene *TBP*, forward primer: 5′‐CACGAACCACGGCACTGATT‐3′ and reverse primer: 5′‐TTTTCTTGCTGCCAGTCTGGA‐3′. *hTERT* mRNA levels were normalized to the expression of the *TBP (TATA‐box‐binding protein)* gene. For Wilms' tumor 1 (*WT1*) gene, qRT‐PCR was performed using WT1 PrimePCR™ SYBR® Green Assay (Bio‐Rad, Des Plaines, IL, USA) according to the manufacturer's instructions. qRT‐PCR analyses were run on a Stratagene Mx3005P System (Agilent Technologies, Santa Clara, CA, USA). Each sample was performed in triplicate, and the mean value was calculated. Results were obtained using the ($2^{‐\Delta \Delta C_{t}}$) method [26]. Values are expressed in arbitrary units (A.U.).

### 2.8 Luciferase assay

Luciferase assays were performed as previously described by Gazon *et al*. [27]. Briefly, 293T cell line was used to set up the protocol, and then, HuT78 and MyLa cells were transfected with a plasmid DNA mixture containing 100 ng·µL^−1^ of pGL3‐hTERT‐378‐Luc reporter plasmid [28], 100 ng·µL^−1^ of pActin‐βgal, and the indicated amount of pAD/WT1‐IRES‐nAMcyan (gift from Edward McCabe, Addgene [Watertown, MA, USA] #29756). HuT78 and MyLa were electroporated using Gene Pulser XCell Electroporation Systems (Bio‐Rad). Forty‐eight hours post‐transfection, cells were washed with cold PBS and then lysed in 1× passive lysis buffer (Promega). Luciferase and β‐galactosidase assays were both performed in a Spark 10M Multiplate Reader (Tecan, Männedorf, Switzerland) with Genofax A Kit and Genofax B Kit (YELEN, Ballaison, France), and Galacto‐Star Kit (Life Technologies, Grand Island, NY, USA), respectively, as described by the manufacturer. Each experiment was performed in triplicate, and Luciferase activities were normalized for transfection efficiency based on β‐galactosidase. After WT1 overexpression, the levels of *hTERT* mRNA were also evaluated by qRT‐PCR.

### 2.9 qRT‐PCR analysis after *WT1* overexpression

Total RNAs were prepared from whole cells using TRIzol (Invitrogen). Briefly, after reverse transcription (RT) using oligo‐dT 12–18 primer (Invitrogen) and SSII reverse transcriptase (Invitrogen), the abundance of transcripts was assessed by real‐time, quantitative PCR analysis using the SYBR Green PCR Master Mix (Roche Diagnostics, Meylan, France) and gene‐specific primer sets. Primer sequences for *hTERT*, hWT1, and *hPRT‐1* are listed in Table S2. Standard curves were generated from each PCR plate for all primer pairs on the plate using a serial dilution of an appropriate experimental sample. Samples were amplified in triplicate on each plate. The conditions for the hTERT PCR were 95 °C for 30 s, 55 °C for 40 s, and 72 °C for 1 min for 45 cycles, while *hWT1* and *hPRT1* were amplified as previously described [29]. Data were analyzed using lightcycler® 480 Software (Roche Diagnostics). Relative mRNA levels of *hTERT* and *hWT1* among experimental samples were determined as previously described [29].

### 2.10 Western blot

Western blot assay was performed according to the manufacturer's recommendations. Briefly, protein extracts from HuT78 cell line, SS PDCs 1, 2, and 3, and MCF7 cell line (positive control expressing WT1, recommended by the manufacturer) in addition to All Blue Prestained Protein ladder (Bio‐Rad) were separated by SDS/PAGE on 8–16% TGX Stain‐Free™ Protein Gels (Bio‐Rad) for approximately 45 min at 150 V in TGX buffer (Bio‐Rad). Stain‐free gels were activated by exposure to UV for 1 min. Proteins were transferred to nitrocellulose membranes (Bio‐Rad) using the Bio‐Rad Trans‐Blot Turbo Transfer System for 7 min. Total proteins on membranes were detected using the stain‐free method. Membranes were blocked with TBST with 5% BSA for 1 h. Membranes were then incubated with primary antibody (WT1 monoclonal antibody, Santa Cruz Biotechnology, Santa Cruz, CA, USA) diluted 1 : 500 in TBST with 5% BSA at 4 °C overnight. Excess of primary antibody was removed by washing the membranes three times in TBST for 10 min each. The secondary antibody (peroxidase‐conjugated anti‐mouse DyLight 800) diluted 1 : 5000 was incubated with the membrane in TBST with 5% BSA for 1 h. Excess of secondary antibody was removed by washing the membranes three times in TBST for 5 min each. Membranes were visualized using Bio‐Rad ChemiDoc™ Imager. Detection and quantification of bands' intensities were done using image lab Software (Bio‐Rad).

### 2.11 *WT1* ChIP‐qPCR assay

*WT1* ChIP‐qPCR assays were performed by Active Motif (Carlsbad, CA, USA). Briefly, *WT1* ChIP‐qPCR assay was performed using 30 μg of chromatin obtained from cultured cells (HuT78, SS PDCs 1, 2, and 3) or primary human T lymphocytes (healthy CD4^+^ cells) and 8 μg of WT1 antibody sc‐192 (Santa Cruz Biotechnology). qPCRs were performed using primer pairs (Table S1) designed for the region of interest (*hTERT*‐323) and for two positive controls (TAL1‐2k and hTERT‐709). A negative control was also used, consisting of a primer pair that amplifies a region in a gene desert on chromosome 12 (Untr12). Data were normalized to the genomic DNA for the particular cell type.

### 2.12 Telomerase activity by TRAP assay

Telomerase activity was assessed in CTCL cell lines and SS PDCs (1, 2, 3, and 4) using the TRAP assay (TRAPeze Telomerase Detection Kit; S7700, Millipore, Alsace, France). Protein extracts were used to extend a synthetic telomeric DNA by PCR amplification (1 cycle of 30 °C for 30 min, followed by a telomeric PCR amplification: 95 °C for 3 min, 2 cycles of 95 °C for 20 s and 49 °C for 20 s, followed by 30 cycles of 95 °C for 20 s and 60 °C for 20 s with signal acquisition) on a Stratagene Mx3005P System (Agilent Technologies). Each sample was run in duplicate with a control DNA.

### 2.13 Statistical analysis

General statistical analyses were performed using the graphpad prism version 5 (San Diego, CA, USA). *P* values of less than 0.05 were considered statistically significant.

## 3 Results

### 3.1 CTCL cell lines and SS patients' tumor cells express *hTERT*

**Fig. 1:**
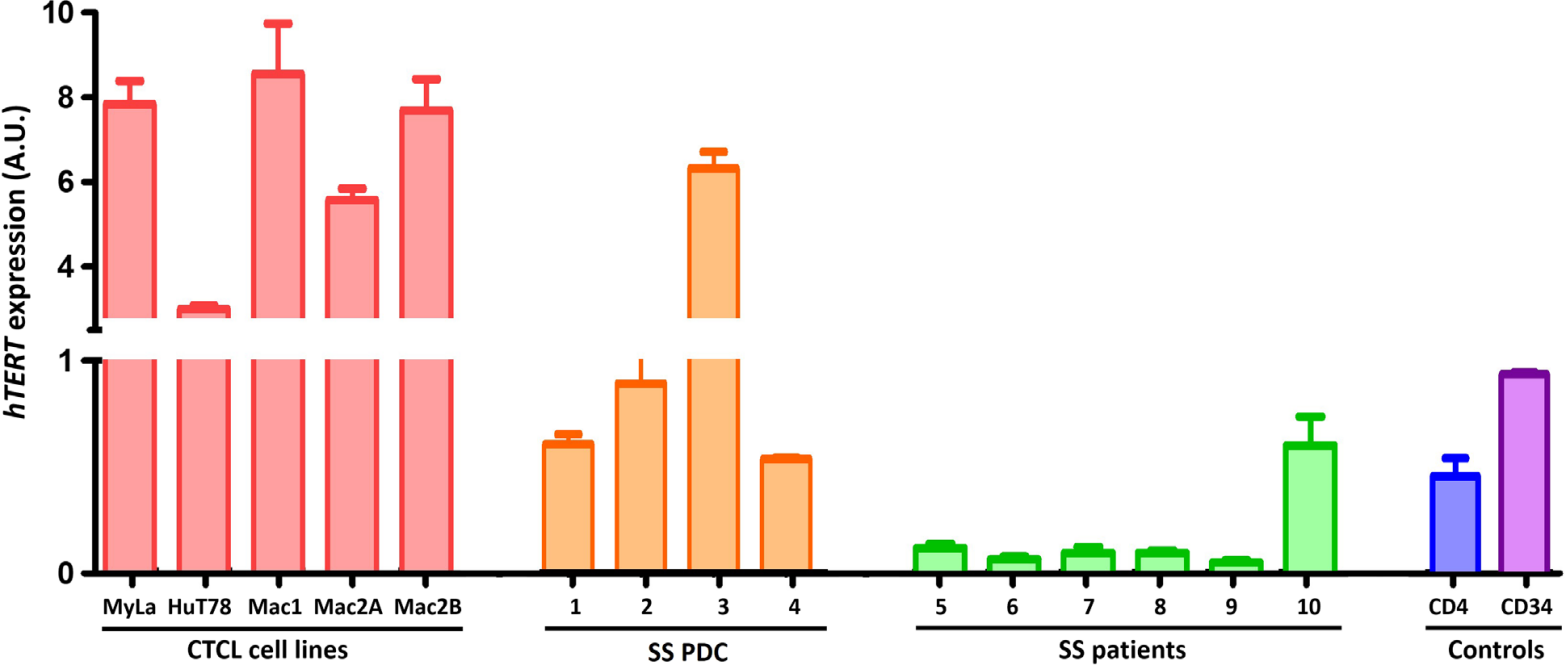
*hTERT* expression in cell lines and patients' cells. *hTERT* mRNA levels quantified by fluorescence real‐time reverse transcriptase PCR in CTCL cell lines, in SS patient‐derived cells (SS PDCs), in SS patient cells (SS patients), and in healthy CD4^+^ and CD34^+^ cells. *hTERT* mRNA levels were normalized to the expression of the *TBP* gene and expressed in arbitrary unit (A.U) ± the SEM of three independent experiments. TBP: TATA‐box‐binding protein located on 6q27. *n* = three independent experiments.

Healthy controls CD4^+^ and CD34^+^ showed *hTERT* expression of 0.47 and 0.95 A.U., respectively (Fig. 1). Compared to healthy controls, CTCL cell lines expressed the highest *hTERT* levels (ranging from 2.7 to 8.2 A.U.) (Fig. 1). In SS PDC, *hTERT* was expressed. While PDC 3 showed *hTERT* expression level similar to cell lines (6 A.U.), PDCs 1, 2, and 4 showed *hTERT* expression levels in the same ranges of those of healthy controls with 0.60, 0.88, and 0.50 A.U., respectively (Fig. 1). In SS patients' fresh cells, *hTERT* was expressed at lower levels (0.07–0.12) than healthy controls (0.47 and 0.95), except for patient 10 with 0.60 A.U. (Fig. 1). Besides, the tumor burden allowed the evaluation of *hTERT* expression in nontumor T cells only in half of the SS patients. No Ct values were obtained for the normal cells in these three patients tested. The *hTERT* expression levels reported, correlated with telomerase activity in CTCL cell lines and in SS PDC with an *R* ^2^ equal to 0.7502 (Fig. S2).

### 3.2 THOR is methylated in CTCL cell lines and SS PDC

**Fig. 2:**
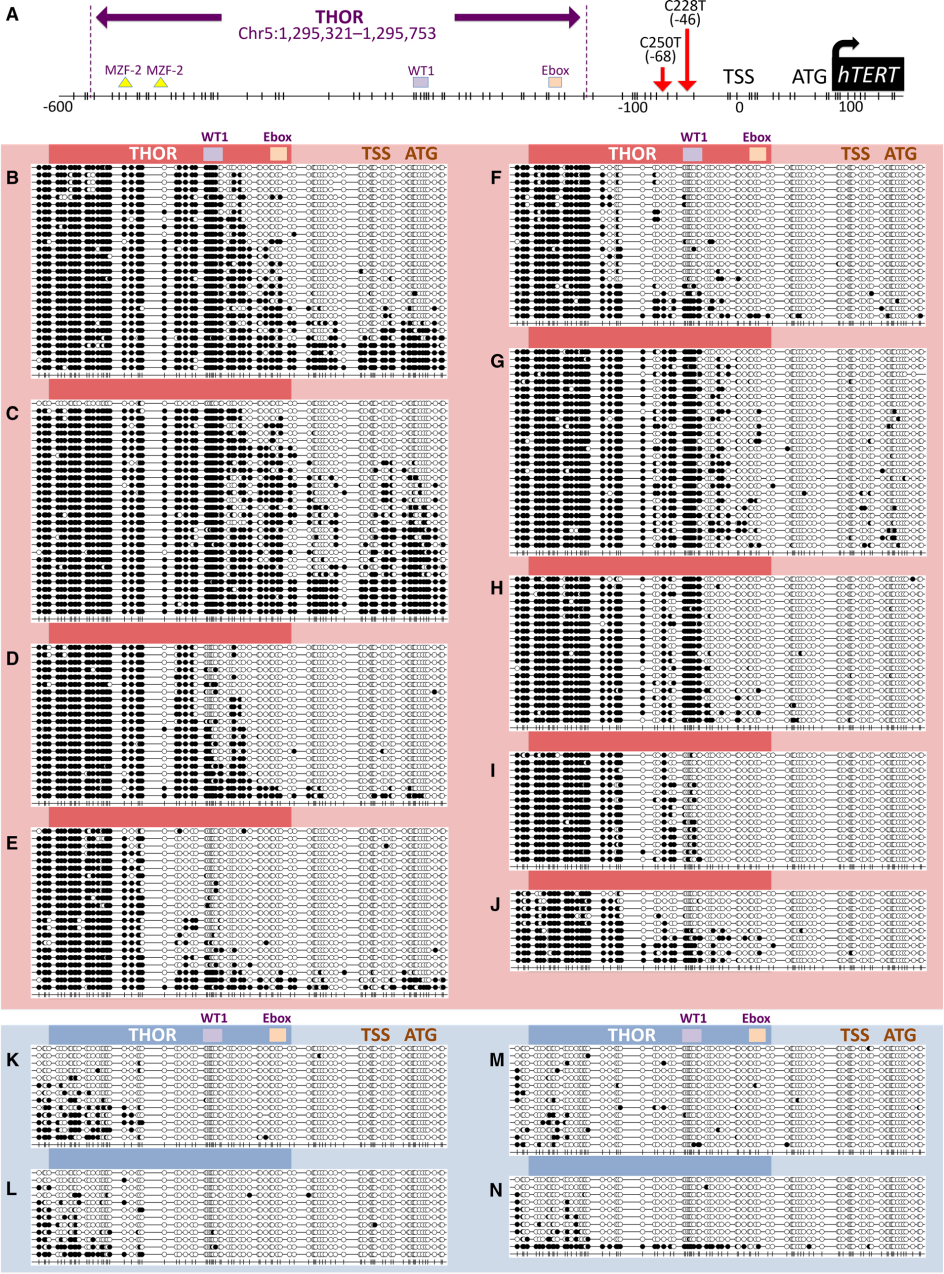
*hTERT* gene promoter methylation including THOR in CTCL cells and healthy controls. (A) *hTERT* gene promoter including THOR (Chr5:1 295 321–1 295 753;GRCh37/hg19) containing 52 CpG represented each by a vertical dash. (B–N) Methylation profiles of CTCL cells (red) and controls (blue): Full black dots represent methylated CpGs, whereas empty dots represent unmethylated CpGs. For CTCL cell lines: MyLa is represented in (B), HuT78 in (C), Mac1 in (D), Mac2A in (E), and Mac2B in (F). For SS PDC: PDC 1 is represented in (G), PDC 2 in (H), PDC 3 in (I), and PDC 4 in (J). Healthy CD4^+^ controls: Two pools are represented in (K) and (L). Normal stem/progenitor cells: Two pools of normal CD34^+^ cells are represented in (M) and (N). *n* = three independent experiments.

A common *hTERT* promoter methylation pattern in CTCL cell lines and in SS PDC was revealed by locus‐specific bisulfite sequencing (Fig. 2). This pattern comprises a hypermethylated distal region between −650 and −150 bp from the TSS, as well as a hypomethylated proximal region between −150 and +150 bp including the TSS and the ATG start codon. The hypermethylated region in CTCL cell lines corresponds to the region recently known as THOR (Fig. 2A). Among CTCL cell lines (Fig. 2B–F), HuT78 presented the highest levels of THOR methylation with an average of 87% (Fig. 2C), followed by MyLa with 83% (Fig. 2B) and Mac1 with 64% (Fig. 2D). Mac2A (Fig. 2E) and Mac2B (Fig. 2F) showed hypermethylation levels around 49% and 45%, respectively. Regarding SS PDCs 1, 2, 3, and 4, hypermethylated THOR levels were 73%, 67%, 50%, and 53%, respectively (Fig. 2G–J). On the contrary, a very low level of methylation was observed in healthy CD4^+^ cells (Fig. 2K,L) and CD34^+^ cells (Fig. 2M,N): 11% and 7.5%, respectively. Interestingly, THOR methylation levels were significantly increased in cell lines and SS PDC compared with healthy cells (*P* < 0.0001). No direct correlation was observed between THOR methylation levels and *hTERT* mRNA levels (Fig. S3).

### 3.3 THOR hypermethylation is a specificity of tumor cells

**Fig. 3:**
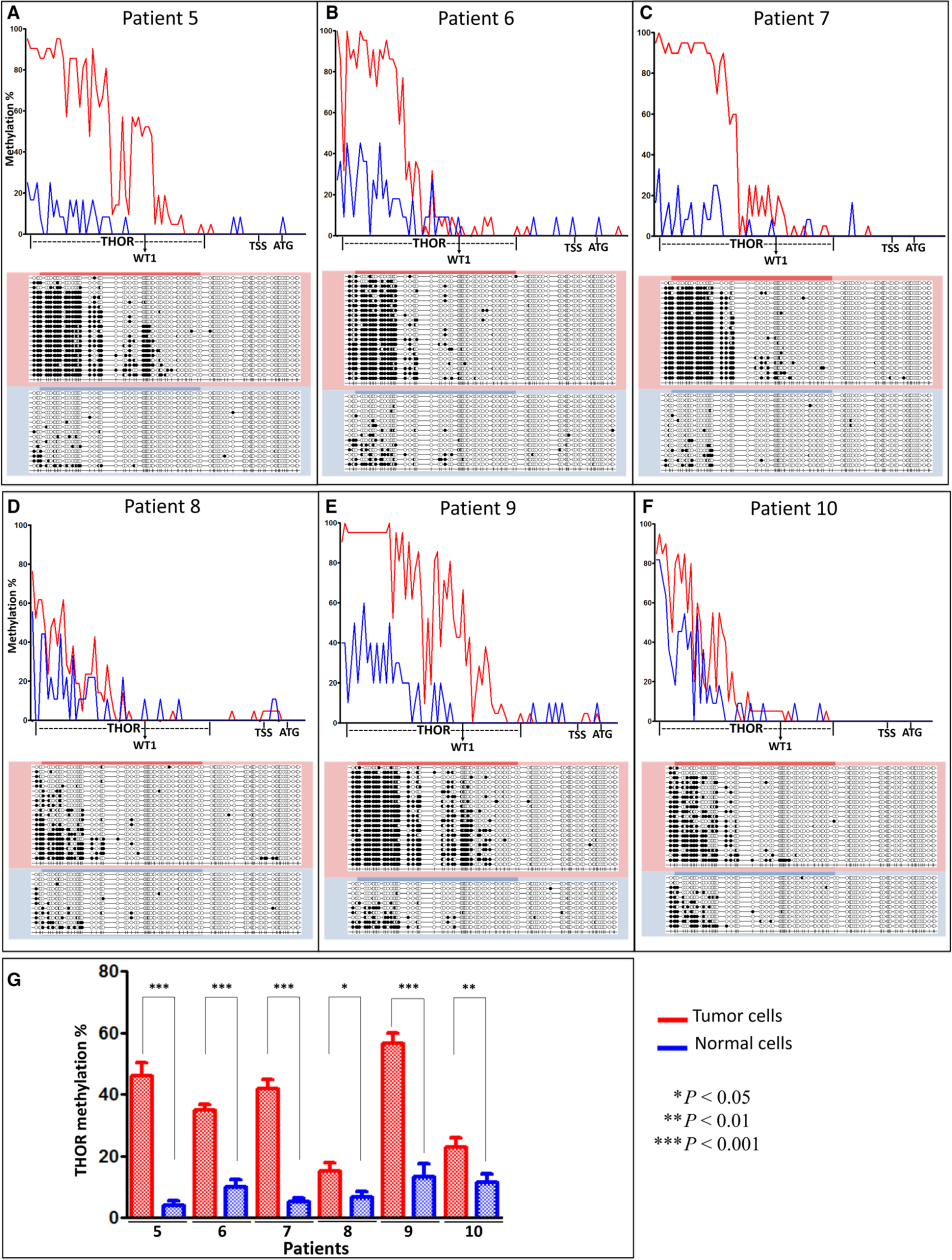
*hTERT* gene promoter methylation including THOR in Sézary syndrome patient cells. Graphs (A) to (F) showing the difference between methylation profiles of tumor cells (red) and normal cells (blue), in patient 5 (A), patient 6 (B), patient 7 (C), patient 8 (D), patient 9 (E), and patient 10 (F). Chart (G) showing THOR methylation levels in tumor (red) and normal (blue) cells in each of the six SS patients' cells (±SEM). Statistical significances were determined by *t*‐test. THOR, TERT hypermethylated oncogenic region.

**Fig. 4:**
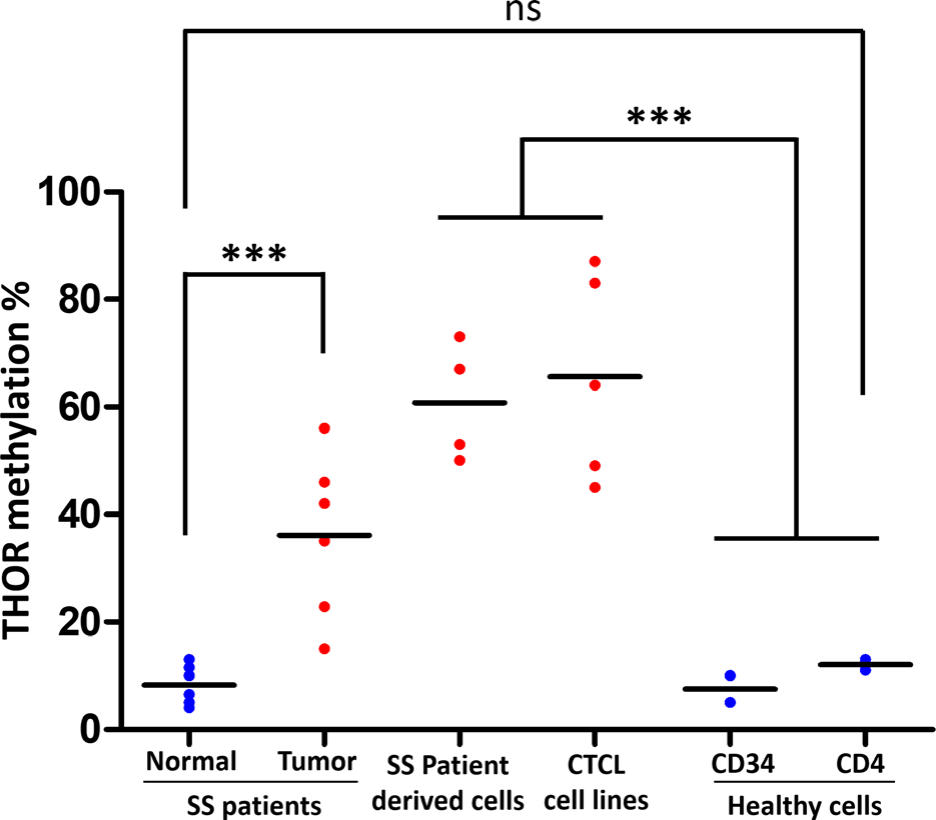
THOR methylation status. The difference in THOR methylation averages between normal cells in blue and tumor cells in red in all cells studied. This figure is a visual representation of THOR methylation in the previously mentioned CTCL cell lines, SS PDC, and SS patients. Statistical significances were determined by *t*‐test. *n* = three independent experiments.

In order to strengthen our findings regarding THOR methylation profiles in cultured CTCL cells, we studied the methylation status of *hTERT* promoter in fresh SS patient cells. For each patient, tumor cells (clonal TCRvβ or CD4‐positive cells) and normal cells used as individual controls (TCRvβ or CD4‐negative cells) were sorted and analyzed. Strikingly, THOR methylation levels were prevalently observed higher in tumor cells than in normal cells. A significant difference (*P* < 0.0001) was observed in patients 5 (Fig. 3A), 6 (Fig. 3B), 7 (Fig. 3C), and 9 (Fig. 3E), with an average methylation level of 46%, 35%, 42%, and 56%, respectively, in tumor cells, compared with 4%, 10%, 5%, and 13%, respectively, in normal cells. A significant difference was also found in patients 8 (Fig. 3D) and 10 (Fig. 3F) (*P* = 0.0455 and *P* = 0.0079, respectively) with lower THOR methylation levels in tumor cells (15% and 22.9%, respectively, for tumor cells; and 6.5% and 11.5%, respectively, for normal cell). Figure 3G summarizes THOR methylation levels in the aforementioned six SS patients. In all healthy cells explored (CD4^+^, CD34^+^, and SS patients' normal cells), THOR was hypomethylated with a methylation level ranging from 4 to 13% (Fig. 4). In our study, a cutoff value of 15% was used for SS patients, which is quite similar to that of 16.1% used by Lee *et al*. [16].

### 3.4 WT1 overexpression reduces *hTERT* activation

**Fig. 5:**
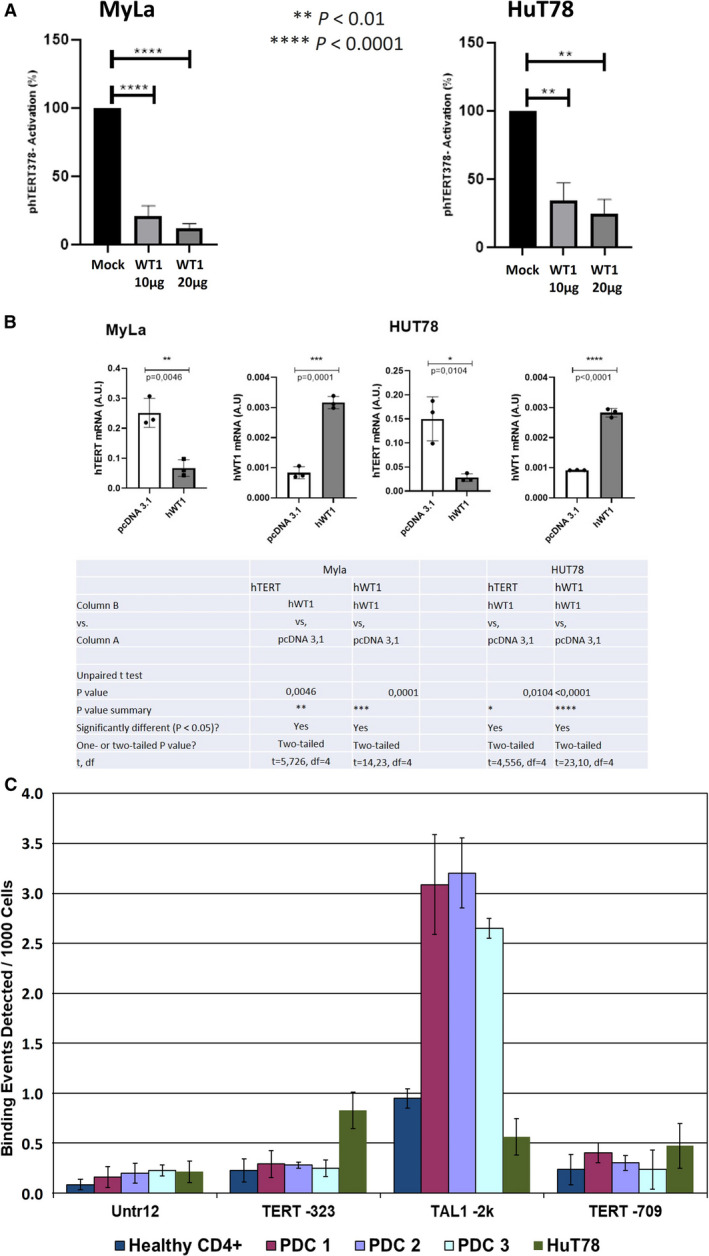
Effect of the transcription factor WT1 on *hTERT* promoter in CTCL. Graph (A) presents the results of luciferase assay showing the effect of empty vector (mock), 10 and 20 µg of WT1 on hTERT promoter activation in HuT78 and MyLa cell lines. Graph and table (B) show the significant decrease in *hTERT* mRNA expression after the overexpression of WT1 in MyLa and HuT78. Graph (C) shows the results of ChIP‐qPCR using a WT1 antibody targeting the TERT‐323 region (region of interest) in SS PDCs 1, 2, and 3, HuT78, a SS cell line, and healthy CD4^+^ (control), along with a negative control (Untr12) region, and two positive control regions (TAL1‐2k and TERT‐709). The results for the TAL1‐2k region confirm the efficacy of the used WT1 primer. Statistical significances were determined by *t*‐test. *n* = three independent experiments. SS PDCs, Sézary syndrome patient‐derived cells; df, degrees of freedom.

Regarding the transcription factors' binding sites on THOR, while the MZF‐2 binding sites were hypermethylated in all tumor samples and the E‐box site was hypomethylated in almost all tumor samples (87%, 13/15 cell lines and patients); WT1 binding site presented different methylation levels between tumor samples (cell lines and patients). For this reason, we focused on WT1 and we assessed by qRT‐PCR the expression levels of WT1 in SS cells (Hut78 cell line and SS PDC). Interestingly, HuT78 and SS PDCs 1, 2, and 3 expressed *WT1* mRNA (Fig. S4A). WT1 protein expression was verified by western blot analysis (Fig. S4B). Next, we evaluated the effect of WT1 overexpression on *hTERT* promoter in two aggressive MF/SS cell lines: MyLa and HuT78. We noticed in these latter that WT1 overexpression reduced significantly the *hTERT* activation in a dose‐dependent manner (Fig. 5A): In MyLa: *P* < 0.0001 with 10 and 20 μg of WT1, while in HuT78: *P* = 0.0051 and 0.0026 with 10 and 20 μg of WT1, respectively. Also, qRT‐PCR analyses showed a decrease in the *hTERT* mRNA levels after WT1 overexpression (Fig. 5B).

### 3.5 WT1 binding on *hTERT* promoter

The obtained results pertaining to the WT1 overexpression and its impact on *hTERT* expression urged us to evaluate the physical interaction between WT1 and *hTERT* promoter. To do so, we used a ChIP‐qPCR approach. Since this assay requires important cells' amounts, we performed it in SS PDC and in a cell line. The SS PDCs 1, 2, and 3 were selected because they derive from epigenetic therapy‐free patients. HuT78 was selected since it was the only SS cell line available in this study. A low value of WT1 binding to *hTERT* promoter region (−323 bp from TSS) was obtained in SS cells (Fig. 5C). This result cannot be due to technical deficiency as no binding events were detected with the negative control primers (Untr12) and significant signals were observed with the positive control primer pair TAL1 (−2k). Regarding normal CD4^+^ cells, we expected to detect WT1 binding on *hTERT* promoter; disappointingly, we observed a faint WT1 binding (−323 bp from TSS). In addition, healthy cells (normal CD4+), HuT78, and PDCs (1, 2, and 3) did not present WT1 binding signals with the additional positive control hTERT‐709.

### 3.6 THOR hypermethylation is insensitive to HDACi

**Fig. 6:**
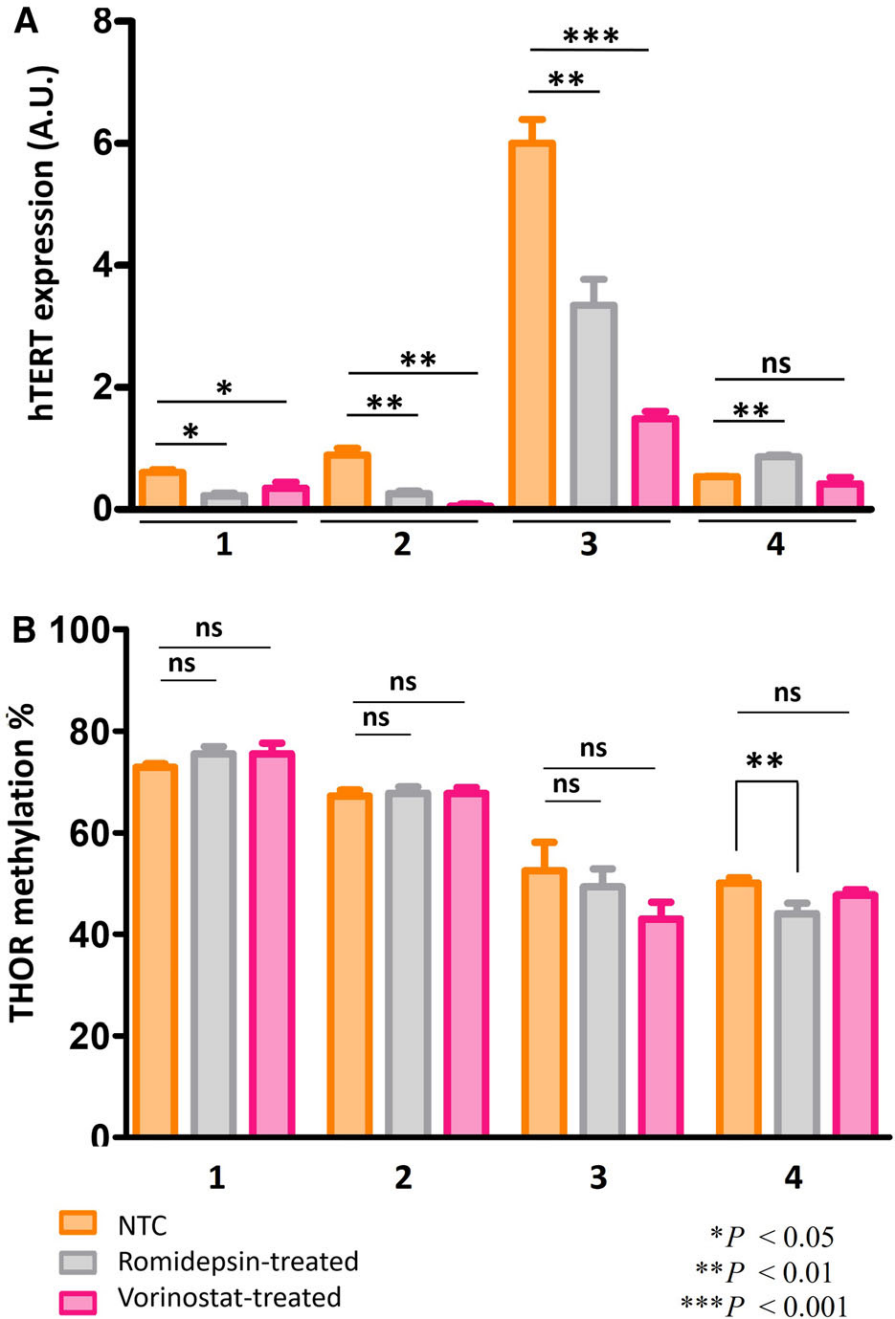
HDACi treatments in Sézary syndrome patient‐derived cells. Graph (A) shows hTERT expression in NTC, romidepsin, and vorinostat‐treated cells. Graph (B) shows THOR methylation % in NTC, romidepsin, and vorinostat‐treated cells (±SEM). Statistical significances were determined by *t*‐test. *n* = three independent experiments. HDACi, histone deacetylase inhibitors; NTC, nontreated cells.

Since two HDACi (romidepsin and vorinostat) are approved for CTCL treatment, without clear molecular investigations, we studied the effect of these two drugs focusing on *hTERT* expression in SS cells. *hTERT* expression decreased significantly (*P* < 0.001) in SS PDCs 1, 2, and 3 after romidepsin and vorinostat treatments (Fig. 6A). Surprisingly, in patient 4, *hTERT* expression was not altered the same way as in the other patients. In fact, *hTERT* expression increased slightly with romidepsin treatment and remained unchanged with vorinostat (Fig. 6A). Methylation levels of *hTERT* promoter in SS PDCs 1, 2, and 3 showed weak changes between nontreated cells (NTC) and cells treated either with vorinostat or with romidepsin (Fig. 6B). Indeed, after HDACi treatments methylation levels and profiles remained quite the same throughout the entire promoter. In patient 4, a slight decrease in THOR methylation status was observed only after romidepsin treatment in comparison with NTC, with a statistical significance of *P* = 0.00063. Overall, following romidepsin or vorinostat treatments, we noticed the absence of particular methylation or demethylation changes at any CpG site (Fig. S5).

### 3.7 THOR hypermethylation is insensitive to 5‐azacytidine

**Fig. 7:**
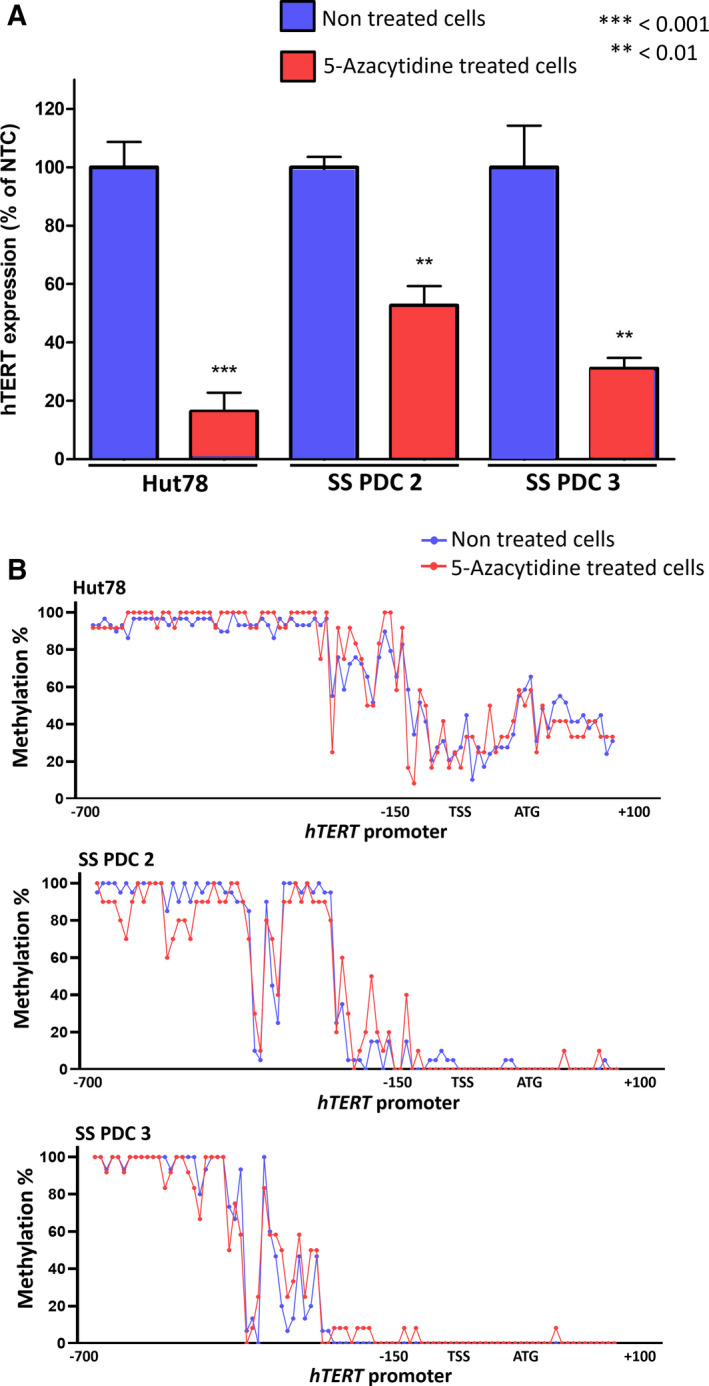
5‐azacytidine treatment in Sézary cells. Graph (A) shows hTERT expression in NTC and 5‐azacytidine‐treated cells (±SEM). Graph (B) shows THOR methylation % in NTC and 5‐azacytidine‐treated cells. Statistical significances were determined by *t*‐test. *n* = three independent experiments. ATG, start codon; NTC, nontreated cells; SS PDCs, Sézary syndrome patient‐derived cells; TSS, transcription start site.

Since HDACi did not exert any effect on THOR methylation in SS PDC, we analyzed the effect of the demethylating agent 5‐azacytidine on *hTRET* expression and promoter methylation in HuT78 cell line and SS PDCs 2 and 3. While *hTERT* expression decreased significantly after 5‐azacytidine treatment in SS PDCs 2 and 3 (*P* < 0.01) and in HuT78 (*P* < 0.001) (Fig. 7A), the methylation status of *hTERT* promoter remained unchanged throughout the entire promoter, showing the same methylation levels and profiles: a highly methylated distal region and a poorly methylated proximal region (Fig. 7B).

## 4 Discussion

Several genetic alterations can result in aberrant *hTERT* expression such as *hTERT* gene amplifications, rearrangements, and promoter mutations [30, 31, 32, 33, 34, 35]. However, in some solid tumors, and in hematological disorders such as non‐Hodgkin lymphomas including CTCL, these known genetic alterations are rare [36, 37]. Additionally, the mechanism responsible for *hTERT* reactivation in these tumors remains unclear. Therefore, several epigenetic mechanisms possibly implicated in *hTERT* promoter regulation were investigated during the last years.

Since *hTERT* promoter DNA is dense in CpG islands (Fig. 2A), numerous studies explored the role of *hTERT* promoter methylation in *hTERT* expression [11, 15, 16, 17]. A recent review of the literature mentioned that *hTERT* promoter does not behave in a simplistic manner [24]. Throughout *hTERT* promoter, the DNA methylation landscape was reportedly not uniform [11, 16, 17]. Two differentially methylated regions were identified: a region containing 52 CpGs located upstream the core promoter and a smaller region around the transcription start site (TSS). In recent investigations led to a variety of cancer cells expressing *hTERT*, the distal promoter region of *hTERT* was recurrently observed hypermethylated [16, 17]. Therefore, *hTERT* distal promoter DNA hypermethylation was associated with *hTERT* re‐expression in cancer cells and proposed as a potential biomarker [7, 12, 15, 16]. This hypermethylated region within *hTERT* promoter was recently named *TERT* hypermethylated oncological region (THOR) [16].

Our previous investigations showed that CTCL cells are *hTERT*‐expressing tumors, while no genetic alterations could explain this expression [19]. In this study, we reported that *hTERT* distal promoter was hypermethylated in all CTCL cells investigated, while the region around the TSS is unmethylated. These observations are in agreement with previous observations in different pathologies [11, 16, 17]. In our study, the comparison between the methylation patterns of *hTERT* promoter in CTCL tumor cells and healthy cells revealed that THOR is unmethylated in healthy CD4^+^ cells and in stem/progenitor cells. This observation underlined that DNA methylation alone is not sufficient to explain *hTERT* expression and that additional epigenetic mechanisms might be implicated [12]. Furthermore, as mentioned by Smith *et al*., aberrant DNA methylation was reported in carcinogenesis across a broad range of cancer types [38, 39]. Therefore, *hTERT* promoter hypermethylation in *hTERT*‐expressing cells represents a characteristic of tumor cells. Thus, nontumor cells expressing *hTERT* might use an alternative epigenetic mechanism to regulate *hTERT* expression. Our current observations in addition to previously published data allowed us to propose that *hTERT* promoter methylation and regulation can be related to the physiological or pathological cell status.

Classically, several binding sites for transcription factors (TFs) either activators (ETS, c‐MYC, SP1, and NFkB) or repressors (WT1 and MZF‐2) are reported within *hTERT* promoter. Among the repressor TFs, we recently questioned the existence of the human MZF‐2 binding sites theoretically located within THOR [40]. In acute promyelocytic leukemia, Azouz *et al*. showed that the methylation of the distal domain of *hTERT* promoter (including THOR) is associated with a decrease in WT1 binding to *hTERT* promoter and sustained *hTERT* expression [11]. As WT1 binding to *hTERT* promoter was reported to be methylation‐sensitive [11], we investigated the role of this binding in SS (advanced‐stage CTCL). First, we verified that WT1 mRNA and protein are expressed in CTCL. Then, we observed that *WT1* overexpression reduced *hTERT* expression. Strengthened by this observation, we investigated the physical interaction between WT1 and THOR in SS cells. Our data suggested that *hTERT* modulation expression in CTCL may occur independently of WT1 binding to the THOR region. However, we are aware that low expressed or low binding levels of some TFs constitute a challenge to be identified. Hence, further investigations are required in order to confirm whether, in CTCL, the binding of this downregulating TF to THOR is methylation‐sensitive and whether other binding TFs might be present in this region.

As reported by Garsuault *et al*., DNA methylation is functionally linked to other epigenetic pathways, including post‐translational histone modifications. This link is mediated by a group of proteins with methyl DNA‐binding activity that localize to methylated DNA and recruit other protein complexes such as histone deacetylases (HDAC) and histone methyltransferases [41, 42]. Since the exact mechanism behind the effectiveness of HDACi treatments in SS patients remains unknown [43], we investigated first the methylation status of *hTERT* promoter after using two HDACi treatments approved in MF/SS patients. Interestingly, after *in vitro* HDACi treatments, *hTERT* expression levels decreased in all SS PDC, while methylation patterns of *hTERT* promoter including THOR remained unchanged, except for one PDC: patient 4, the only patient who had previously received romidepsin. This observation may suggest a possible drug resistance mechanism. In the other patients, THOR remained hypermethylated and *hTERT* proximal promoter encompassing TSS and ATG remained hypomethylated. In a previous study using vorinostat in non‐small cell lung cancer, Li *et al*. observed a repression of the telomerase expression and a reduction in *hTERT* methylation levels near the TSS (around −200 to +160 bp), but THOR was not investigated [44]. In our study, the TSS region was already hypomethylated and rationally cannot be more demethylated. Altogether, these data suggest that HDACi reduced *hTERT* expression only in patients who did not receive previous epigenetic therapies. Besides, we proved that other epigenetic drugs such as 5‐azacytidine, a demethylating agent, did not exert a demethylation on *hTERT* promoter including THOR in SS, while it reduced *hTERT* expression. In other pathologies, it has been reported that *hTERT* expression decreased after treatments with demethylating agents. This decrease could be accompanied by a promoter demethylation, suggesting that *hTERT* promoter demethylation could be cell type and pathology‐dependent [45, 46]. It has been also reported that demethylating drugs can exert different effects on different genes. In some genes, this effect is ‘direct’ with a decrease in the methylation levels accompanied by a corrected gene expression, whereas in other genes named ‘refractory’, DNA methylation and gene expression remain unchanged under demethylating drug pressure [47, 48, 49, 50]. Nevertheless, another group of genes respond to the demethylating drugs through some changes in gene expression, while their promoter methylation levels remain unaltered. This mechanism, termed ‘indirect’, can happen through the demethylation of a TF controlling the specific gene expression, or the demethylation of regulatory elements such as enhancers, or by a secondary response to DNA damage or reparation mechanisms, or also through histone modifications. Altogether, our results suggest that *hTERT* promoter methylation in CTCL is resistant to the direct effect of epigenetic drugs, indicating that these drugs can alter *hTERT* expression in an indirect way.

## 5 Conclusions

Since the mechanism behind *hTERT* expression in CTCL remains unknown, we undertook the first epigenetic study of *hTERT* promoter in this pathology. Overall, our findings strongly suggest that THOR hypermethylation is a hallmark of neoplastic CTCL cells associated with *hTERT* activation. Additionally, we propose that THOR hypermethylation might be used as a biomarker of cancer cells in SS patients. By adding CTCL to the list of tumors analyzed for THOR methylation, our findings represent a significant step forward toward a better understanding of the mechanisms involved in telomerase activation and its regulation by epigenetic therapies in this pathology. Moreover, our data provide a starting point for further investigations to assess the relationships between THOR methylation status, *hTERT* expression, and TF binding with THOR in order to fully understand the sophisticated molecular mechanism of *hTERT* activation in CTCL. The advent of new gene‐specific targeting tools [20] will help to establish causality between *hTERT* promoter DNA methylation and *hTERT* expression, paving the way to a better understanding of the clinical response to epigenetic drugs in advanced‐stage CTCL patients.

## Conflict of interest

The authors declare no conflict of interest.

## Author contributions

AC performed DNA/RNA/protein extractions, bisulfite sequencing, tumor cell isolation, western blot, and statistical analyses, analyzed and interpreted the data, wrote the manuscript, and prepared the figures; AC, JR, JF, and YI performed qPCR analyses; JF performed TRAP assay; AC, SP, MP‐C, and FC performed flow cytometry analyses, cell cultures, and HDACi/DNMTi treatments; JMP performed luciferase assay and WT1 overexpression; MBB and APL recruited SS patients and followed them up; ES‐B, J‐MP, JPM, MB‐B, ElC, CF, and RT provided helpful advices and assisted in editing the manuscript; EdC conceived and designed the study, and revised and edited the final version of this manuscript. All authors read and approved the final version of the manuscript.

### Peer Review

The peer review history for this article is available at https://publons.com/publon/10.1002/1878‐0261.12946.

## Supporting information


**Fig. S1.** Evaluation of tumor cells proportion before and after cell sorting.
**Fig. S2.** Correlation between *hTERT* expression level and telomerase activity.
**Fig. S3.** Absence of correlation between THOR methylation status and hTERT expression level.
**Fig. S4.** WT1 mRNA and protein expression.
**Fig. S5.** hTERT promoter methylation profiles after HDACi treatments.
**Table S1.** *hTERT* Bisulfite PCR conditions and primer sequences.
**Table S2.** Primer sequences for *hTERT*, *hWT1* and *hPRT‐1*.
**Table S3.** Primer sequences used for *WT1* ChIP‐qPCR.

## Data Availability

The data that support the findings of this study are available on request from the corresponding author. The data are not publicly available due to privacy or ethical restrictions.
