# Supplementary material for: Exploring hTERT promoter methylation in cutaneous T‐cell lymphomas
Source: Mol Oncol. 2021 Oct 12;16(9):1931–46. doi: 10.1002/1878-0261.12946 (PMC9067155; doi:10.1002/1878-0261.12946)
Supplement: Supplementary file 1 — Fig. S1. Evaluation of tumor cells proportion before and after cell sorting. Fig. S2. Correlation between hTERT expression level and telomerase activity. Fig. S3. Absence of correlation between THOR methylation status and hTERT expression level. Fig. S4. WT1 mRNA and protein expression. Fig. S5. hTERT promoter methylation profiles after HDACi treatments. Table S1. hTERT Bisulfite PCR conditions and primer sequences. Table S2. Primer sequences for hTERT, hWT1 and hPRT‐1. Table S3. Primer sequences used for WT1 ChIP‐qPCR. [file MOL2-16-1931-s001.pdf]

Supplementary table 1: *hTERT* Bisulfite PCR conditions and primers sequences

**Forward Primer**	5’ GGTTTGTGTTAAGGAGTTTAAGT 3’	
**Reverse Primer**	5’ CCAACCCTAAAACCCCAAAC 3’	
**PCR program**	2 min: 94° C	
	30s: 94° C<br>30s: 58° C<br>1min: 72° C	35 cycles
	5min: 72° C	

Supplementary table 2: Primer sequences for *hTERT*, *hWT1* and *hPRT-1*

**qRT-PCR**	**Forward Primer**	**Reverse Primer**
hTERT	CGGAAGAGTGTCTGGAGCAA	GGATGAAGCGGAGTCTGGA
hWT1	CCAGCTTGAATGCATGACCTG	GCCCTTCTGTCCATTTCACTG
HPRT1	TGACACTGGCAAAACAATGCA	GGTCCTTTTCACCAGCAAGCT

Supplementary table 3: Primer sequences used for *WT1* ChIP-qPCR

**Region**	**Primer sequence**	**Purpose**
hTERT -323 -F	AGCGGAGAGAGGTCGAATC	Region of interest
hTERT -323 -R	AGGGCCTCCACATCATGG	
hTAL1 -2k -F	CAGAAGGGCAGCAAACAAAC	Positive control
hTAL1 -2k -R	GTGTCCTGTTGGGCAGTGTG	
hTERT -709 -F	GAGCAAACCACCCCAAATC	Positive control
hTERT -709 -R	TCCATTTCCCACCCTTTCTC	
Untr12	Active Motif Human negative control primer set 1, #71001	Negative control

A

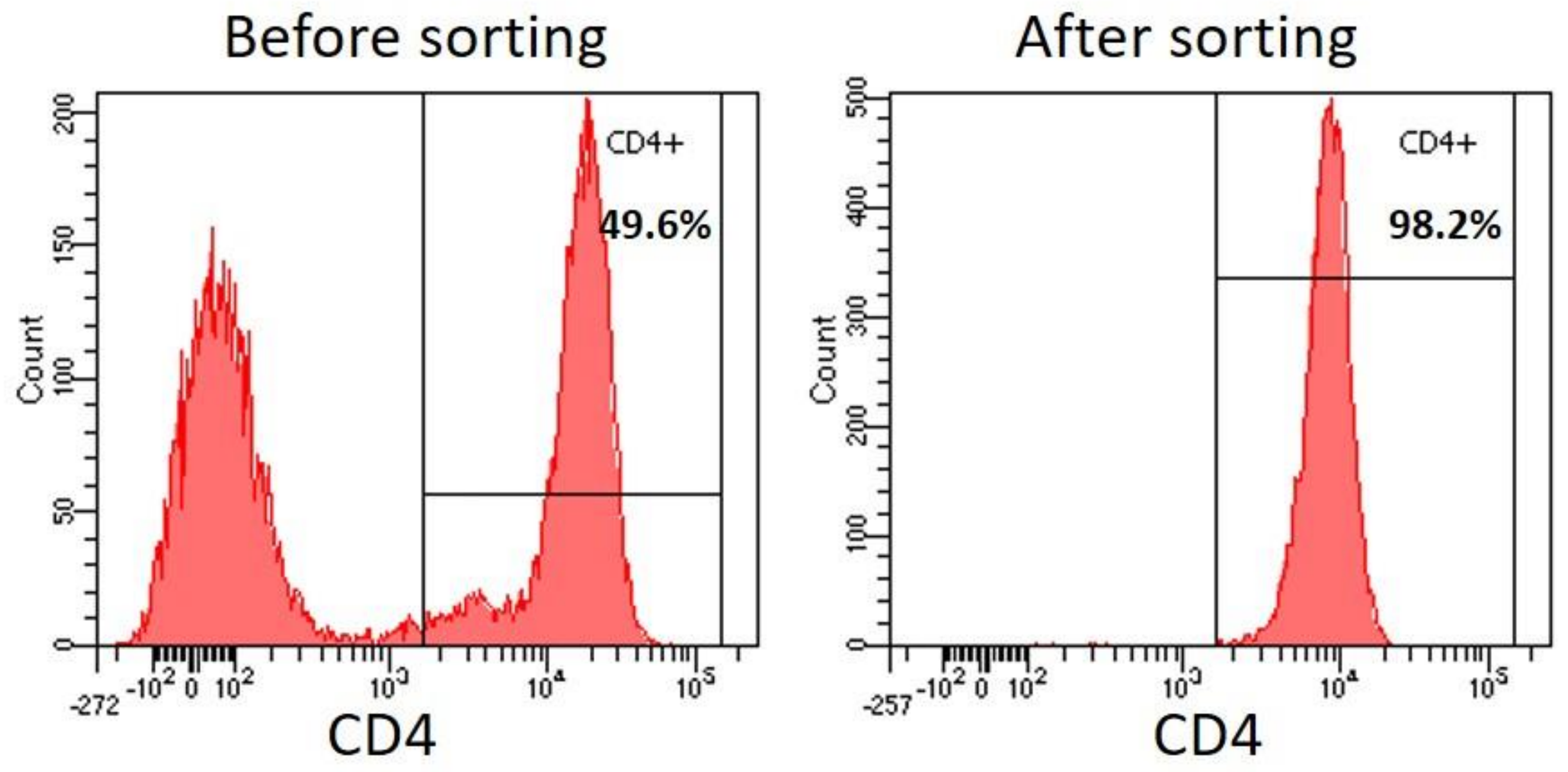

B

Patients	% of CD4+ or TCRVB+ cells in before sorting	% of CD4+ or TCRVB+ cells after sorting
5	49.6% (CD4)	98.2%
6	41.8% (TCRV8)	97.0%
7	21.5% (TCRVB17)	97.1%
8	25.9% (CD4)	98.7%
9	50% (CD4)	99.2%
10	30.3% (CD4)	99.3%

Supplementary figure 1: Evaluation of tumor cells proportion before and after cell sorting

Fresh cells were isolated by magnetic or flow sorting by the selection of CD4+ or TCRVβ+ population when it was possible. (A) Example of histogram showing the proportions of the tumor cells before and after sorting. (B) Table summarizing the proportions of the tumor cells before and after sorting for all patients.

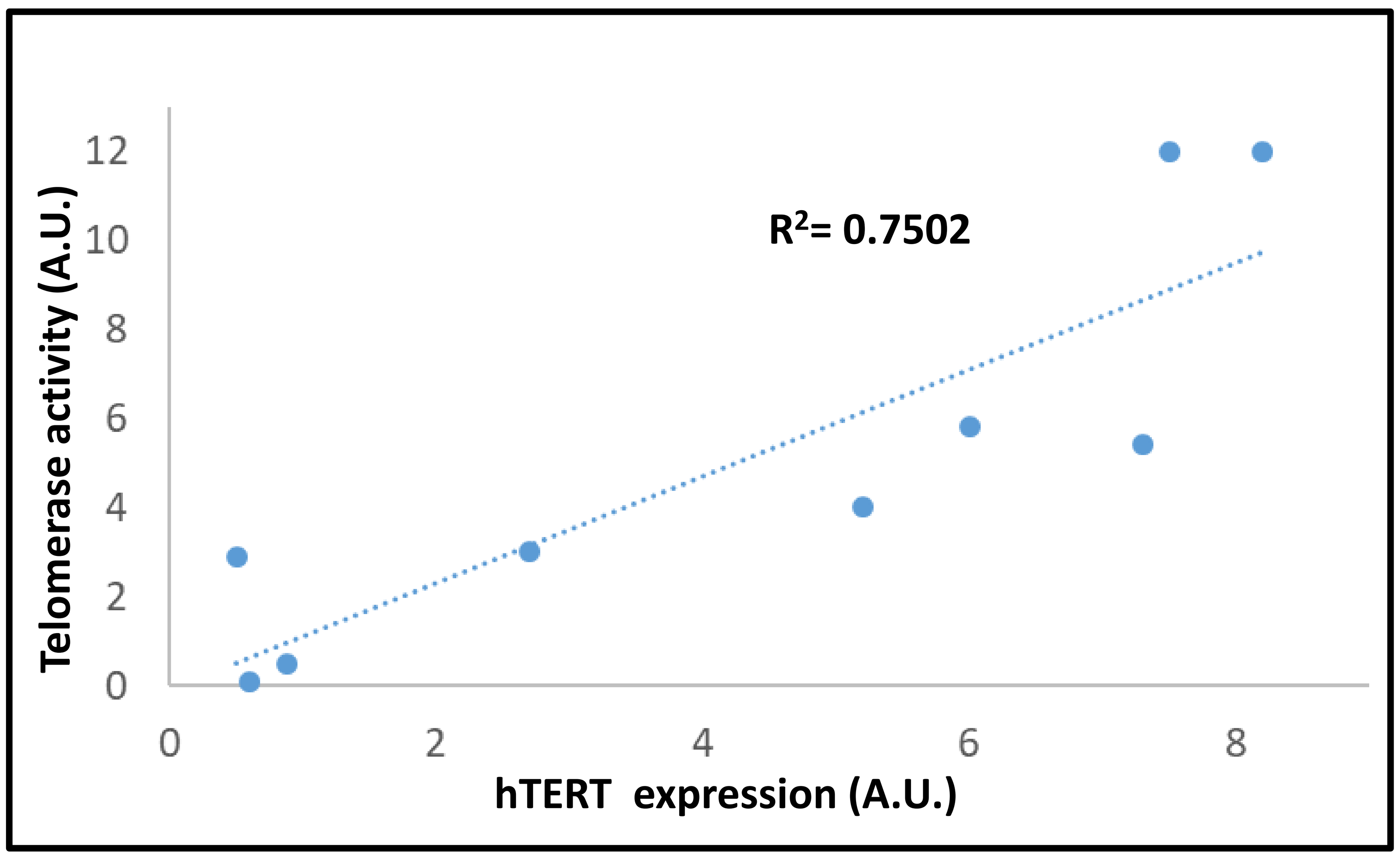

Supplementary figure 2: Correlation between hTERT expression level and telomerase activity.

hTERT mRNA levels and telomerase activity evaluated by TRAP assay are correlated in CTCL cell lines and SS PDC with R2 = 0.7502. CTCL: Cutaneous T-Cell Lymphomas; SS PDC: Sézary Syndrome Patients-Derived Cells. .

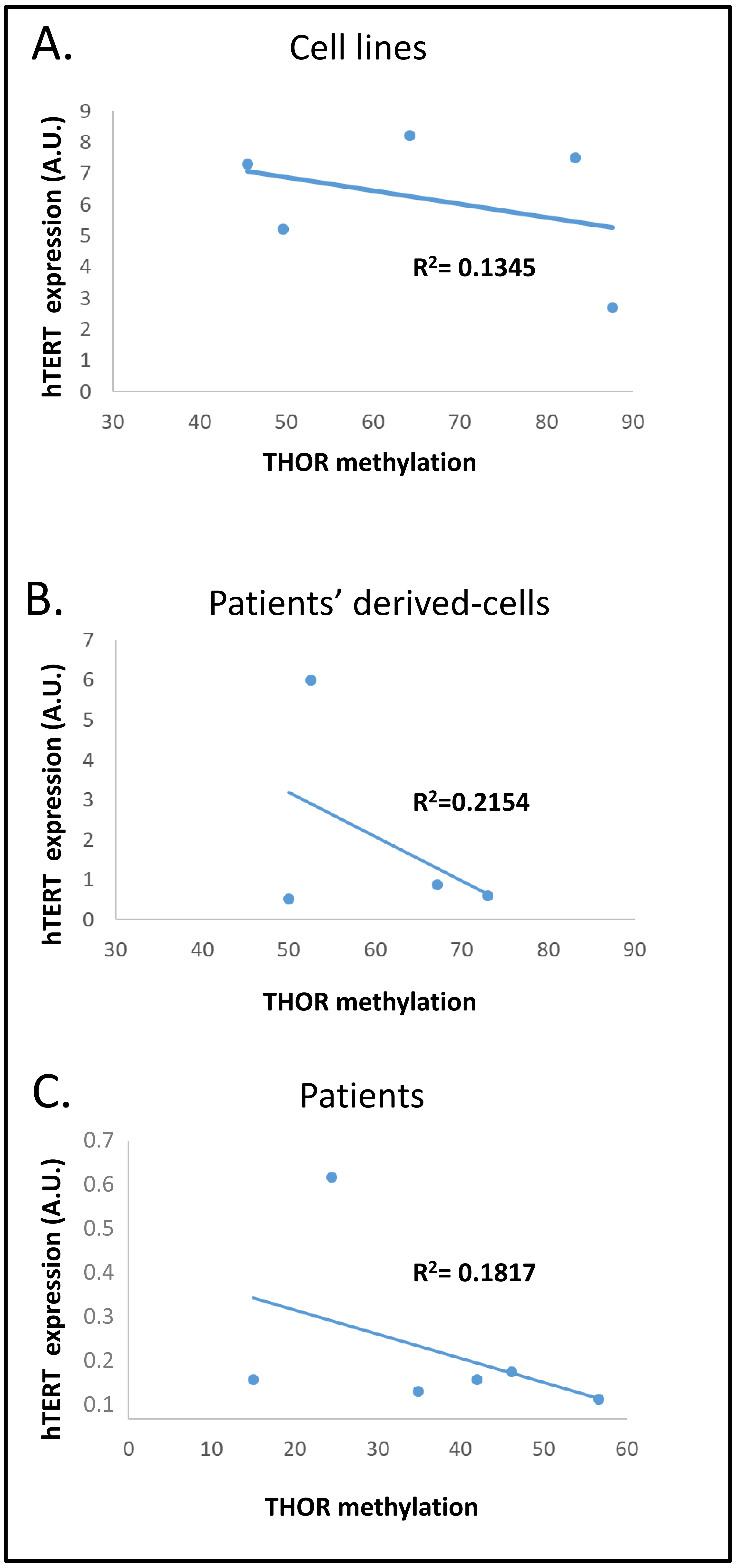

Supplementary figure 3: Absence of correlation between THOR methylation status and hTERT expression level.

R2 coefficients showing the absence of correlation between THOR methylation status and hTERT expression level in CTCL cell lines (A), SS PDC (B) and SS patients (C). CTCL: Cutaneous T-Cell Lymphomas; SS PDC: Sézary Syndrome Patients-Derived Cells; SS: Sézary Syndrome.

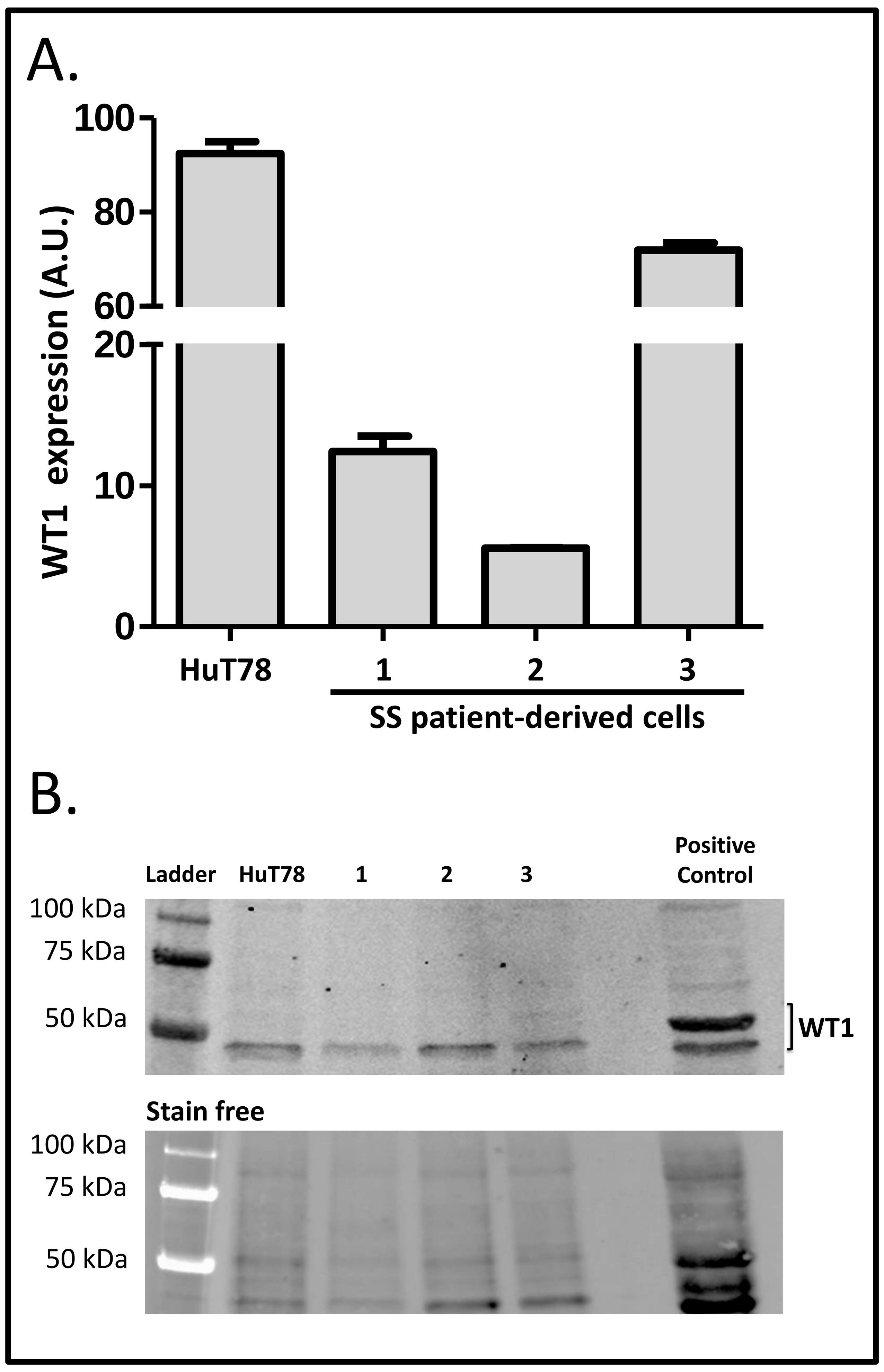

Supplementary figure 4: WT1 mRNA and protein expression.
(A) WT1 mRNA levels normalized to the expression of the TBP gene and expressed in arbitrary unit (A.U.) and (B) WT1 protein levels by western blot in HuT78 cell line, in SS PDC 1, 2 and 3 and in MCF7 as positive control using the stain-free technology. SS PDC: SS PDC: Sézary Syndrome Patients-Derived Cells.

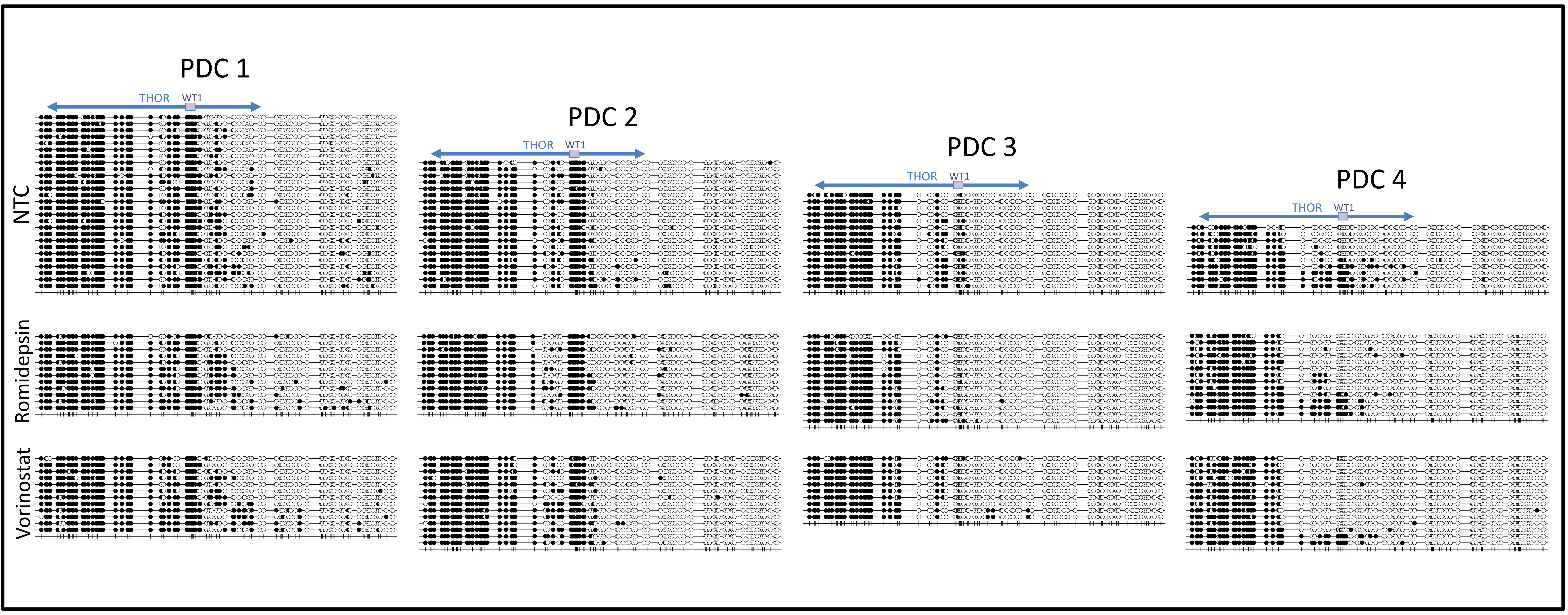

Supplementary figure 5: hTERT promoter methylation profiles after HDACi treatments.
hTERT promoter methylation profiles of SS PDC 1, 2, 3 and 4 in NTC and in romidepsin or vorinostat –treated cells. HDACi: Histone DeACetylases inhibitors; SS PDC: SS PDC: Sézary Syndrome Patients-Derived Cells, NTC: Non-Treated Cells.
